# Implementing health communication tools at scale: mobile audio messaging and paper-based job aids for front-line workers providing community health education to mothers in Bihar, India

**DOI:** 10.1136/bmjgh-2021-005538

**Published:** 2021-07-21

**Authors:** Victoria Ward, Safa Abdalla, Hina Raheel, Yingjie Weng, Anna Godfrey, Priyanka Dutt, Radharani Mitra, Padmapriya Sastry, Sara Chamberlain, Melissa Shannon, Kala Mehta, Jason Bentley, Gary L Darmstadt, MD, Ananya Study Group, Yamini Atmavilas

**Affiliations:** 1 Department of Pediatrics, Stanford University School of Medicine, Stanford, California, USA; 2 Quantitaitve Sciences Unit, Department of Medicine, Stanford University, Stanford, California, USA; 3 BBC Action Media, London, UK; 4 BBC Media Action, New Delhi, Delhi, India; 5 Department of Epidemiology and Biostatistics, University of California San Francisco, San Francisco, California, USA

**Keywords:** child health, maternal health, paediatrics, prevention strategies, public health

## Abstract

**Introduction:**

As part of an investment by the Bill & Melinda Gates Foundation to support the Government of Bihar to improve reproductive, maternal, newborn and child health and nutrition (RMNCHN) statewide, BBC Media Action implemented multiple communication tools to support front-line worker (FLW) outreach. We analyse the impacts of a package of mHealth audio messaging and paper-based job aids used by FLWs during government-sponsored village health, sanitation and nutrition days (VHSNDs) on knowledge and practices of childbearing women across the RMNCHN continuum of care.

**Methods:**

Data from two surveys collected between July and September 2016 were analysed using logistic regression to compare health-related knowledge and behaviours between women who had been exposed at VHSNDs to the mHealth GupShup Potli (GSP) audio recordings or interpersonal communication (IPC) tools versus those who were unexposed.

**Results:**

Exposure to GSP recordings (n=2608) was associated with improved knowledge across all continuum-of-care domains, as well as improved health-related behaviours in some domains. The odds of having taken iron-folic acid (IFA) tablets were significantly higher in exposed women (OR 1.5, 95% CI 1.1 to 2.2), as was contraceptive use (OR 2.0, 95% CI 1.2 to 3.2). There were no differences in birth preparedness or complementary feeding practices between groups. Exposure to IPC paper-based tools (n=2002) was associated with a twofold increased odds of IFA consumption (OR 2.3, 95% CI 1.7 to 3.2) and contraceptive use (OR 1.8, 95% CI 1.2 to 2.8). Women exposed to both tools were generally at least twice as likely to subsequently discuss the messages with others.

**Conclusion:**

BBC Media Action’s mHealth audio messaging job aids and paper-based IPC tools were associated with improved knowledge and practices of women who were exposed to them across multiple domains, suggesting their important potential for improving health outcomes for beneficiaries at scale in low-resource settings.

**Trial registration number:**

NCT02726230.

**Key questions**

**What is already known?**

- Well-trained and supported front-line workers (FLWs) can expand the reach and quality of health education and services in low resource settings.
- The modern proliferation of mobile technologies has created new opportunities for improved communication and broader dissemination of key health information.

**What are the new findings?**

- We studied the impacts of a package of mHealth and paper-based communication tools intended to empower FLWs in their delivery of health information at significant scale (28 million population) in a low-resource setting.
- Exposure to the tools was associated with significantly improved knowledge of key health information and reported health-related behaviours when compared with those who were not exposed to them. Exposure to the tools was also associated with increased discussions among beneficiaries and others in their community.

**Key questions**

**What do the new findings imply?**

- mHealth-based communication tools have the promise to significantly improve FLW communication with beneficiaries, as well as increased knowledge and uptake of key health behaviours.
- The use of paper-based visual demonstration tools was associated with improved health-related knowledge and behaviours among beneficiaries, suggesting that their use can engender understanding of new and complex concepts.
- These findings suggest important new opportunities for mechanisms of delivery of critical health messages, involving dissemination of educational information at significant scale through mobile technology to women who may not otherwise have access to facility-based healthcare.

## Introduction

The thousand days between conception and an infant’s second birthday is a highly vulnerable period requiring critical health interventions to promote optimal growth and development and to combat the major threats to morbidity and mortality. Yet despite the clear evidence for effective solutions to improve health for pregnant mothers and infants in this period—such as antenatal care, birth preparedness practices, skilled birth attendance, early and exclusive breast feeding and skin-to-skin care—reductions in morbidity and mortality have fallen short of global goals.^1^ Impacts of interventions may be attenuated by the challenges of maintaining their quality and coverage at scale.^2 3^ This is particularly true in rural and under-resourced areas where there is often a severe shortage of trained and supervised healthcare workers to implement them^4^ and transportation to healthcare facilities may be limited. Thus, women in these areas may have poor access to care, including key reproductive, maternal, newborn and child health and nutrition (RMNCHN) services.^5^

A growing body of research has suggested that a primary solution for improving access to quality healthcare in low-resource settings is to expand front-line worker (FLW) delivery of health education and services. It has also been shown that training and supportive supervision are critical to ensure successful delivery of reliable and well-communicated health messages.^6 7^ To provide this support and to further expand quality healthcare for marginalised populations in hard-to-reach places, mobile health (mHealth) services have been employed to empower FLWs and their beneficiaries with novel methods of education and communication. In the past decade, there has been a rapid expansion of mobile phone technology available throughout low-income and middle-income countries (LMICs), creating a new opportunity to support the delivery of educational information by providing interactive voice response (IVR) and short message service (SMS)-based training via mobile phones. Health training can be developed using engaging and scientifically vetted educational information which can be implemented at remarkable scale quickly and cost-effectively to reach diverse geographies, ethnicities and languages.

Multiple systematic reviews have assessed the impacts of mHealth interventions in low resource settings, suggesting that the use of mobile technology for clinical decision support and FLW training has led to substantial improvements in the quality of care provided.^8 9^ Our studies of mHealth tools supporting FLW delivery of RMNCHN services in Bihar, India, showed significant improvements in quality and frequency of service delivery, leading to improved health behaviours of beneficiaries.^10 11^ Furthermore, mobile-delivered education, when designed using an equity lens, can provide life-saving information in even the hardest to reach and otherwise media-dark areas.^12^

While the utilisation of mHealth tools is promising, particularly in low resource areas where access to information and health services may be limited, it is critical that they be designed with a human-centred approach and subsequently evaluated to assess the benefit for knowledge and health-related behaviours.^13^ In this study, we assess ‘facilitated communication’ tools by evaluating the impact of a package of mHealth and paper-based FLW job aids to determine if their use was associated with improved key RMNCHN knowledge, greater interpersonal discussion, and improved behaviours among childbearing women in Bihar, India.

## Methods

### Study setting

In the past 10 years, India has consistently had some of the highest burdens of maternal and child deaths annually. Among its poorest states is Bihar, a 38 400 square mile area in the northeast of the country with over 100 million people. Here, unstable infrastructure and high crime rates resulted in some of the worst maternal and child health indicators of any Indian state (online supplemental table 1). However, it also has had a quickly growing economy and increasing government attention to maternal and child health outcomes.

10.1136/bmjgh-2021-005538.supp1 Supplementary data

In 2010, the Bill & Melinda Gates Foundation (BMGF) partnered with the government of Bihar (GoB) to improve statewide RMNCHN health outcomes through Ananya, a large-scale technical support programme. Their investment supported the implementation of interventions to expand the availability of quality health services while simultaneously building demand for them through improved health knowledge and behaviours. New tools and interventions were designed and piloted by grantees, with subsequent provision of technical support to the GoB to facilitate statewide scale-up of those found to be successful.^14^ In order to achieve impact at scale, a grant entitled ‘Shaping Demand and Practices’ was awarded in 2011 to the organisation BBC Media Action to develop a large-scale, multiplatform suite of interventions across the eight innovation districts, reaching 28 million people, with plans to scale up effective interventions across all 38 districts of Bihar in 2014.^15^ In this study, we evaluate the impacts of a package of mHealth and paper-based job aids for use by FLWs in group settings, to assess whether they improved health-related knowledge, subsequent interpersonal discussion and behaviours among beneficiary women.

### Interventions

One approach of the Shaping Demand and Practices grant within the Ananya programme was to increase demand for health services through education and empowerment of healthcare workers promoting specific health behaviours.^11^ Community-based interventions were implemented at village health, sanitation and nutrition days (VHSNDs), an initiative of the Indian government to provide monthly community events during which FLWs would provide health information and services to women and children. BBC Media Action developed supportive tools for FLWs working at these VHSNDs, with the intention of reinforcing their counselling of women with quality health information. GupShup Potli (GSP) was one such service, a mobile phone-based audio recording of twelve modules covering key health content about a variety of RMNCHN behaviours. FLWs connected their phones to speakers using an auxiliary cord, and played recordings for beneficiaries. The recordings were then followed with a facilitated discussion and a question and answer session on health-related topics for the group of women with whom they spoke.^16^ A second set of tools also utilised by FLWs at VHSNDs were the interpersonal communication (IPC) tools. These physical job aids included four main demonstration modules to explain to groups of women key health messages regarding diarrhoea management, iron-folic acid (IFA) consumption, immunisation compliance, birth spacing and contraception utilisation. The job aids were used to provide visual demonstrations of key health information during the conversations with groups of women. These facilitated communication tools (ie, tools that support FLW communication), were created using principles of human-centred design.^15^

### Implementation

Similar to other interventions of the Ananya programme,^14^ BBC Media Action initially piloted its tools during 2014 and 2015, starting in the eight focus districts and expanding to an additional seven districts for the paper-based tools. In order to reach beneficiaries statewide, implementation would be reliant on the transfer of ownership and implementation to the GoB to scale up effective interventions to the rest of the 38 districts alongside the supply-side interventions of CARE India.^17^

### Data sources

In this study, we evaluated the impact of the VHSND communication tools developed and delivered by BBC Media Action in partnership with the GoB and other implementing partners. To do so, we undertook a secondary data analysis of multiple cross-sectional surveys collected between July and September 2016 providing comparable data on the health-related knowledge and behaviours across the continuum of maternal and child care.

These surveys were implemented by interviewers trained and supervised by the organisation, Market Sapience. Questionnaires used logic checks, skips and ranges which were scripted for Computer Administered Personal Interviews using tablet computers.

### Sampling methodology

For both studies, the primary sampling unit was the block level. Out of the 137 blocks in the eight intervention (or focal) districts, 13 intervention blocks were chosen through random selection for the GSP survey and 7 blocks for the IPC survey. Similarly, 13 blocks from the non-intervention (or nonfocal) districts were chosen, matched across demographic and key health indicators. Within each selected block, 10 gram panchayats or clusters of villages, were randomly selected. Within the gram panchayats, a listing was conducted to identify eligible women for inclusion who were either currently pregnant or had recently had a child up to 1 year of age. From these, five pregnant women and five mothers of children less than 1 year old were randomly selected from each group. Women were excluded if they had not attended at least one of the VHSNDs in the preceding 3 months. Given that not all women within the intervention areas were exposed to the GSP or IPC tools, as the decision to use them was at the discretion of FLWs, self-reported recall of the tools having been used to deliver messages was used as the exposure variable. Respondents who had attended a VHSND but reported that they had not been exposed to the tool were shifted to the unexposed group, regardless of whether they were selected within focal or non-focal districts. Thus, while sampling areas were selected randomly, the study design was not experimental, rather the knowledge and self-reported health-related behaviours were compared between those who were exposed to the interventions and those who were not using a post-test comparison of groups. Women who were exposed to the GSP or IPC tools were also asked if they discussed what they had learnt from the FLW after the VHSND session with anyone and, if so, with whom did they speak. Thus, exposed women were further subdivided into those who had discussed a range of topics and those who had not discussed these topics with anyone else.

### Statistical analysis

For maternal respondents surveyed across the data sets, a standardised set of sociodemographic characteristics were compared across the respondents who reported that they were exposed to the interventions as compared with those respondents who did not report exposure to interventions. Baseline demographic characteristics with significant differences between the exposed and unexposed groups were later adjusted for in the analysis, including age, religion, caste and birth order. Among those who recalled each particular topic, a sub-analysis examined whether they had discussed the topic with anyone else afterward, comparing those who were exposed versus unexposed after adjusting for sociodemographic covariates that were significantly associated with the exposure in each topic group. P values were calculated using two-sample t-tests for the continuous variables and χ^2^ tests for categorical variables. Fisher’s exact tests were conducted for categorical variables with small cellular frequency in any sub-group. Health behaviours were estimated by logistic regression modelling using self-reported exposure to the health message via the GSP or IPC tool as the primary independent variable. Of those who had recently attended a VHSND, those women who reported exposure to the GSP or IPC tool regarding a specific topic were compared to those who were unexposed, respectively. Percentages were reported as crude percentages without adjusting for any survey design or weights. ORs with respective 95% CIs were reported for each indicator. All analyses were assessed at alpha=0.05, and all were conducted in Stata V.14^18^ and SAS V.9.4.^19^ No imputation was used for missing data and all data were handled as complete-case analysis.

### Patient and public involvement

The initial survey design used in this study relied on questionnaires developed to elicit the knowledge and behaviours of individual programme beneficiaries. Pilot testing of questionnaires was carried out to ensure acceptability of the survey. Neither patients nor the public were involved in the data analysis, reporting or dissemination of this research.

### Role of the funding source

This study was supported by grants from the BMGF, including: OPP1163688 to Stanford University, OPP1084426 to CARE India and OPP1017359 to BBC Media Action. The senior author had full access to the data and independence from the funders in the reporting of results, the interpretation of the data and the decision to publish the manuscript.

## Results

### Demographics

A total of 2608 women were surveyed for the GSP study and 2002 women for the IPC study. Demographics of the cohorts surveyed are described in table 1 by study area but not subcategorised by exposure since this varied for each indicator. The numeric breakdown of those who reported exposure for each message is described in online supplemental table 2.

**Table 1:** Demographic characteristics of the maternal household respondents in surveys used to evaluate the GupShup Potli and interpersonal communication tools as part of the Ananya programme in Bihar, India (July–September 2016)

Maternal characteristics	VHSND GSP	VHSND IPC
**Sample size (n**)	2608	2002
**Age in years (mean, SD**)	24.7 (4.3)	24.7 (4.3)
**Religion**		
Hindu	79.5	75.6
Muslim	19.6	23.5
Others	0.8	1.0
**Caste**		
Scheduled caste/tribe	29.2	30
Other backward class	56.4	57.1
General caste	8.3	7
Others	6.1	5.9
**No formal schooling**	54.2	54.7
**Birth parity**		
No other children	14.5	14.9
1 child	26.3	26.6
2 children	25.6	24.6
3 children	18.1	17.8
4+ children	15.4	16.1

GSP, GupShup Potli; IPC, interpersonal communication; VHSND, village health, sanitation and nutrition days.

#### GupShup Potli

Among maternal respondents who reported exposure to the GSP mobile phone-based audiorecordings, health topic recall was significantly higher across all messages when compared with those who were unexposed (table 2).

**Table 2:** Recall of particular health topic discussed during VHSND sessions in the preceding 3 months attended by maternal respondents who were exposed versus unexposed to the GupShup Potli tool as part of the Ananya programme in Bihar, India

Health message	Unexposed (n=1736)	Exposed (n=872)	P value*
	%	%	
Growth monitoring	3.5	9.5	<0.001
Pneumonia	8.5	22.8	<0.001
Birth preparedness	22.6	32.8	<0.001
Complimentary feeding	26.1	41.3	<0.001
Antenatal visits	19.2	28.7	<0.001
Birth spacing	2.7	21.1	<0.001
Diarrhoea management	0.3	5.7	<0.001
Immunisation	5.3	21.4	<0.001
Don’t remember	37.7	7.9	<0.001

*All models adjusted for religion, caste and no formal schooling.

VHSND, village health, sanitation and nutrition day.

With regard to knowledge and behaviours surveyed, for those who were exposed to the GSP tool, the odds of having appropriate knowledge and behaviours related to indicators across the continuum of care were compared with those who were unexposed (table 3). Knowledge was significantly higher among women exposed to GSP compared with those unexposed when asked about birth preparedness (OR 1.3, 95% CI 1.0 to 1.7, p=0.03), tetanus toxoid (TT) vaccines (OR 1.5, 95% CI 1.0 to 2.1, p=0.03), complementary feeding (OR 1.6, 95% CI 1.2 to 2.2), growth monitoring (OR 1.8, 95% CI 1.1 to 2.8) and pneumonia care (OR 4.9, 95% CI 1.8 to 13.1) (table 3). Women who were exposed were more likely to take IFA tablets (OR 1.5, 95% CI 1.1 to 2.2) and were twice as likely to be currently using contraception (OR 2.0, 95% CI 1.2 to 3.2), however, there was no difference in plans for future use of contraception (OR 1.0, 95% CI 0.7 to 1.4). There was no difference in the practice of complementary feeding, or in receipt of the tetanus vaccine. Women who were exposed were less likely to have had their children receive immunisations (OR 0.6, 95% CI 0.4 to 0.9).

**Table 3:** Assessing the impact of GupShup Potli interventions on the knowledge and practice of targeted behaviours among maternal household respondents exposed to corresponding health messages as part of the Ananya programme in Bihar, India

	Unexposed*	Exposed*	OR (95% CI)†
	%	%	
Consumption of iron folic acid (IFA)	35.2	45.9	1.5 (1.1 to 2.2)
Knowledge of growth monitoring	55.5	68.7	1.8 (1.1 to 2.8)
Knowledge of birth preparedness‡	53.2	59.1	1.3 (1.0 to 1.7)
Practice of birth preparedness activities (currently pregnant women)§	82.8	88	1.6 (0.9 to 2.5)
Knowledge of complementary feeding¶	74.8	83.3	1.6 (1.2 to 2.2)
Initiation of complementary feeding at 6 months (mothers of children <12 months)	56.7	62.3	1.3 (0.9 to 1.8)
Knowledge of pneumonia care**	91	98	4.9 (1.8 to 13.1)
Knowledge of tetanus toxoid (TT) vaccine¶	76	82.8	1.5 (1.0 to 2.1)
Practice of TT vaccine (currently pregnant women)	91.6	92.4	1.4 (0.7 to 2.9)
Practice of immunisations (mothers with at least one child)	82.4	73.6	0.6 (0.4 to 0.9)
Plans to use contraception	39.6	41.5	1.0 (0.7 to 1.4)
Current use of contraception (non-pregnant women)	12.2	22.7	2.0 (1.2 to 3.2)

*N for each indicator described in online supplemental table 2.

†All models adjusted for age, caste, religion and birth order.

‡Birth preparedness knowledge is defined as positive response on the importance of >2 of the following: registration of pregnancy, identification of an institution for delivery, saving money for delivery, saving numbers for FLW, saving numbers for ambulance, or arranging transport for delivery. The lower bound of the CI was rounded down from 1.02 to 1.0 for consistency, p=0.03.

§Positive practice of birth preparedness is defined as a woman completing >2 of the above.

¶Knowledge of complementary feeding is defined as the response that complementary feeding should be started at 6 months of age.

**Knowledge of TT vaccine is defined as understanding that a woman should receive 2 TT vaccines.

FLW, front-line worker.

### IPC tools

Women exposed to the IPC tools were twice as likely to report both correct knowledge and practice of IFA compliance than those who had not been exposed (table 4). Women exposed to the diarrhoea tool were three times as likely to know how to use ORS and zinc to treat diarrhoea as compared with the unexposed (OR 3.0, 95% CI 2.3 to 4.1), however, the practice of appropriate management was not significantly different. While the knowledge of birth spacing was not significantly different between groups, both the current use (OR 1.8, 95% CI 1.2 to 2.8) and plans to use contraception (OR 1.4, 95% CI 1.1 to 1.8) were more likely among the exposed group.

**Table 4:** Assessing the impact of the interpersonal communication tool on the knowledge and practice of targeted behaviours among maternal household respondents exposed to the corresponding health message as part of the Ananya programme in Bihar, India

	Unexposed*	Exposed*	OR (95% CI)†
	%	%	
Knowledge of iron folic acid (IFA)‡	32.9	47.6	1.9 (1.5 to 2.4)
Current use of IFA (currently pregnant women)	41.4	61.9	2.3 (1.7 to 3.2)
Knowledge of diarrhoea management§	10.0	24.6	3.0 (2.3 to 4.1)
Use of oral rehydration solution or zinc if diarrhoea occurred	67.4	75.0	1.7 (0.6 to 4.9)
Knowledge of birth spacing	64.5	64.9	1.0 (0.8 to 1.3)
Plan to use contraception	37.1	46.3	1.4 (1.1 to 1.8)
Current use of contraception	10.2	17.5	1.8 (1.2 to 2.8)

*N for each indicator described in online supplemental table 3.

†All models adjusted for age, caste, religion and birth order.

‡Knowledge of IFA is defined as a positive attestation to >2 of the following benefits of usage: (a) supplementation of blood production in the body, (b) improved growth and development of the unborn child, (c) birth of a healthy child, (d) reduction in the chances of complications during pregnancy and childbirth.

§Knowledge of diarrhoea management is defined the understanding that a child should be given zinc and oral rehydration solution in the case of symptomatic presentation.

### Interpersonal discussions

Overall, women exposed to the GSP tool were at least twice as likely to have discussed the health topic across nearly all content areas with someone following exposure (table 5), most commonly with their husbands, family members or friends (online supplemental table 4). Women who were exposed to the IPC tool were twice as likely to discuss the topics of IFA tablet consumption and diarrhoea management, but there was no difference in subsequent discussions about birth spacing between the two groups (online supplemental table 5).

**Table 5:** Comparison of those who reported having had discussions about topics they had heard about at VHSND sessions among women who were exposed vs unexposed to the Gsp tool

Topic*	% Unexposed (N)	% Exposed (N)	OR (95% CI)†
Growth monitoring	60.7 (56)	79.0 (81)	2.4 (1.1 to 5.4)
Pneumonia	50.0 (136)	74.5 (192)	2.9 (1.8 to 4.8)
Birth preparedness	52.6 (365)	68.7 (275)	1.8 (1.3 to 2.6)
Complementary feeding	48.9 (415)	70.2 (346)	2.5 (1.9 to 3.5)
Antenatal care checkups	47.8 (322)	72.8 (243)	3.1 (2.1 to 4.4)
Birth spacing	31.8 (44)	69.9 (173)	5.0 (2.4 to 10.2)
Diarrhoea management	100 (4)	82.6 (46)	NA
Immunisation	46.2 (78)	70.4 (179)	2.8 (1.6 to 5.0)

*Missing a valid response: growth monitoring: 6, pneumonia: 18, birth preparedness: 38, complementary feeding: 52, ANC check-ups: 18, birth spacing: 13, diarrhoea management: 5, immunisation: 22.

†Adjusted for age, caste, religion and birth order.

ANC, antenatal care; NA, not available; VHSND, village health, sanitation and nutrition day.

## Discussion

In the studies of both the GSP and IPC interventions implemented by BBC Media Action during VHSNDs, significant differences were observed in the knowledge and health-related behaviours of women who were exposed to the tools compared with those were not. Women who were exposed to the mHealth GSP content were significantly more likely to recall health messages and were also more likely to report knowledge and healthy behaviours associated with that information. They were also more likely to report appropriate IFA consumption and current use of contraception, as well as improved knowledge about birth preparedness, TT vaccine administration, growth monitoring, complementary feeding and pneumonia care. They were less likely, however, to have had their children receive immunisations, likely in part because most women primarily attended VHSND events for the purpose of obtaining vaccines and therefore, their intention to practice was already high. The content related to preventive benefits of immunisations was also thought to be too technical, and the messages about disease avoidance not immediate enough to motivate behaviour change. Women exposed to the IPC tools during VHSND sessions were more likely to report appropriate knowledge and health practices for IFA consumption and contraception usage, as well as improved knowledge of diarrhoea management. There was no difference, however, in the use of ORS for diarrhoea. For both tools, exposed women were at least twice as likely to subsequently discuss most health topics with another person, such as their husband, relative or friend.

Overall, this study demonstrated that exposure to BBC Media Action’s mHealth-based VHSND tools was associated with a range of improvements in RMNCHN-related knowledge and practices, as well as discussions by beneficiaries with others regarding the topics. These findings align with the growing evidence for the role of mHealth solutions in improving RMNCHN programmes in LMICs. Many previous studies of mHealth tools have shown targeted benefits, particularly in ANC compliance and breastfeeding practices.^20–22^ However, they have less often demonstrated benefits for postnatal care practices such as immunisation compliance and contraception usage.^23–25^ Yet while the evidence is mounting for the benefit of digital solutions that provide direct-to-beneficiary communications, such as SMS reminders for ANC behaviours or stage-specific educational recordings,^9^ or those that target improved workflows for FLW healthcare delivery,^10 26 27^ far fewer rigorous studies have assessed the impacts of mHealth solutions for facilitated communications tools for FLW–beneficiary interactions. Our findings suggest that digital solutions for facilitated communications reflect the known benefits of using job aids for patient communications in amplifying the impacts of FLW-beneficiary interactions.^11 28^ Information, however, must be effectively designed and rigorously evaluated for its benefit in the target population. For instance, information that is too technical such as that regarding ORS therapy for diarrhoea or the mechanisms of disease prevention from immunisations, may be ineffective in driving behaviour change as was demonstrated by the lack of impact in these domains in our results. Finally, while it has been suggested that the most significant impacts from mHealth are seen in those programmes that combine mHealth and non-mHealth interventions,^29 30^ further study is required to evaluate the domains and behaviours which are most likely to be impacted by digital compared with non-digital tools, as well as potential synergism between them, with additional qualitative assessment of beneficiary experiences of these interventions.

This study was limited in that all surveys were reliant on maternal self-report, and thus exposed to both recall and social desirability biases. Further, they were collected cross-sectionally when multiple other interventions were being implemented as a part of the Ananya programme in the same geospatial areas.^14 31^ While the study design allows for the assumption that women would have had an equal chance of having been exposed to other initiatives, it cannot be ruled out that women who attended FLW-facilitated discussions may be more likely to also attend other educational events, such as radio programmes and street theatre. Further, it is difficult to differentiate whether the benefits for women who were exposed to the tools were due to the content of the tools or simply the confounding effect of having been exposed to an FLW, thus making it difficult to attribute impact to specific interventions. Due to the study design, we were not able to compare the digital versus the non-digital tools with regard to their benefit for knowledge or behaviour change. Additionally, while sampling areas were selected randomly, the study design was not experimental and there may have been selection bias in intervention exposure when FLWs chose those beneficiaries for whom they would use job aid tools. Importantly, the study design was such that no sampling weights were applied. Finally, because intensive support and facilitation were provided for FLWs by BBC Media Action during the implementation period, generalisability may be limited given the challenges for sustainability and scalability of interventions bolstered by such extensive support.

As the use of mHealth tools becomes an increasingly common mechanism for the delivery of health services and information across LMICs, rigorous evaluations of their impacts are critically important, particularly for implementation at scale. The Shaping Demand and Practices programme of BBC Media Action was intended to improve RMNCHN knowledge and practices across eight focus districts and approximately 28 million people,^32^ with subsequent scale-up statewide to more than 100 million using mobile-based job aids and health messaging tools in Bihar. While the challenges of the government-supported statewide scale-up of these interventions are described elsewhere,^14 31^ implementation in the eight focal districts alone required the training of over 110 000 FLWs. Thus, the scale of implementation and the rigorous evaluation across multiple datasets was unique in its contribution to the literature on mHealth.^11^

## Conclusion

As technology advances and smart phones become more ubiquitous, even in hard-to-reach places, a rich opportunity has emerged for the delivery of quality healthcare through mHealth interventions. Our analysis has shown that implementation of digital and non-digitally facilitated communication tools for FLW support are associated with higher levels of self-reported knowledge and healthy behaviours, as well as subsequent discussions with others in the family. Further study is required, however, to understand how mHealth tools can be utilised most effectively, and in what contexts. For instance, assessments should address whether digital health tools are more effective than paper-based visualisation tools, particularly for supporting less educated or marginalised groups; or when and for what topics digitally facilitated communication is superior to direct-to-beneficiary digital tools. Only then can digital and mHealth assessments be optimised in their evidence-based use for purpose. Importantly, future evaluations of the effectiveness of mHealth interventions must also focus on health outcomes, and the long-term sustainability of health-related improvements. Technology tools continue to create opportunity for improved health impacts at scale, but their use must be evidence-based to ensure cost-effective implementation and sustained benefits for their beneficiaries.

## Data Availability

Data are available, including survey interview guides, from the senior author upon reasonable request through a data sharing agreement with Stanford University and BBC Media Action.
