# Supplementary material for: Implementing health communication tools at scale: mobile audio messaging and paper-based job aids for front-line workers providing community health education to mothers in Bihar, India
Source: BMJ Glob Health. 2021 Jul 21;6(Suppl 5):e005538. doi: 10.1136/bmjgh-2021-005538 (PMC8728373; doi:10.1136/bmjgh-2021-005538)
Supplement: Supplementary data [file bmjgh-2021-005538supp001.pdf]

Supplemental Tables

Supplemental Table 1: National Family Health Survey [NFHS-3 (2005/2006) and NFHS-4 (2015/2016)] data for India (nationwide) and Bihar

Indicator	NFHS-3 (2005-2006) Bihar	NFHS-4 (2015-2016) Bihar	NFHS-3 (2005-2006) India	NFHS-4 (2015-2016) India
Female literacy (age 15-49)	37.0%	49.6%	55.1%	68.4%
Contraception prevalence rate	34.1%	24.1%	56.3%	53.5%
4 or more ANC visits	11.2%	14.4%	37.0%	51.2%
Institutional delivery rate	19.9%	63.8%	38.7%	78.9%
Fully immunised children, ages 12-23 months	32.8%	61.7%	43.5%	62.0%
Children under 6 months exclusively breastfed	28.0%	53.5%	46.4%	54.9%
Iron-folic acid tablet consumption for 100+ days	6.3%	9.7%	15.2%	30.3%

Source: National Family Health Survey, India: http://rchiips.org/nfhs/

Supplemental Table 2: The number of maternal respondents who reported having been exposed versus unexposed to the GSP tool for health message delivery during a recent VHSND.

**Health Message**	**Exposed**	**Unexposed**
Consumption of iron folic acid (IFA)	157	1128
Knowledge of growth monitoring	83	2525
Knowledge of birth preparedness	286	2322
Practice of birth preparedness activities (currently pregnant women)	166	1128
Knowledge of complementary feeding	360	2248
Initiation of complementary feeding at 6 months (mothers of children <12 months)	138	582
Knowledge of pneumonia care	199	2409
Knowledge of tetanus toxoid (TT) vaccine	250	2358
Practice TT vaccine (currently pregnant women)	156	1120
Practice of immunisation (mothers with ≥1 child)	121	1221
Plans to use contraception	159	2275
Current use of contraception (non-pregnant women)	110	1202

Supplemental Table 3: The number of maternal respondents who reported having been exposed versus unexposed to the IPC tool for health message delivery during a recent VHSND.

**Health Message**	**Exposed**	**Unexposed**
Consumption of iron folic acid (IFA)	416	1586
Current use of IFA (currently pregnant women)	215	756
Child had an instance of diarrhoea in the last 3 months (among women with children ≤ 12 months)	215	786
Knowledge of diarrhoea management	387	1615
Use of ORS or zinc if diarrhoea occurred	24	89
Knowledge of birth spacing	333	1492
Plan to use contraception	320	1564
Current use of contraception	183	817

Supplemental Table 4. Amongst those women who reported having discussed the health topic they had heard about via the GSP tool at VHSND sessions with another person, comparison of those with whom they spoke about the topic.

	Growth monitoring	Pneum-onia	Birth prepared-ness	Compli-mentary feeding	Antenatal check-ups	Birth spacing	Diarrhoea manage-ment	Immuni-sation
Sample size (n)	98	211	381	446	331	135	42	162
With whom did you discuss (%)								
Husband	66.3	63.5	70.9	62.5	62.2	62.2	66.7	69.8
Mother-in-law	49.0	49.8	46.7	43.3	54.4	42.2	47.6	50.6
Friend/neighbour	29.6	36.5	28.6	30.7	30.5	26.7	19.1	22.8
Other family member/relative	20.4	19.4	26.8	25.3	22.9	23.7	21.4	22.8
AWW	8.2	1	3.7	3.1	3.9	5.2	4.8	2.5
ASHA	3.1	1	3.2	3.1	4.2	3.7	7.1	3.1
ANM	0	0	1.1	0.9	0.9	0.7	0	0
Someone Else	0	0	0	0	0	0	0	0
Don't know/ Don't remember	3.1	2.4	0.8	97.3	1.2	0.7	4,8	1.2

AWW = Anganwadi worker, ASHA = Accredited Social Health Activist, ANM = Auxiliary Nurse Midwife

Supplemental Table 5. Those who reported having had discussion regarding a topic they had learned about following attendance at a VHSND, comparing women who had been exposed to the IPC tool with those who were unexposed.

**Topic***	**% Unexposed (N)**	**% Exposed (N)**	**OR (95% CI)** †
IFA	64.3 (199)	77.8 (167)	2.1 (1.3 – 3.3)
Diarrhoea management	65.8 (79)	78.7 (89)	2.0 (1.0 – 4.1)‡
Birth spacing	74.4 (156)	75.9 (145)	1.2 (0.7 – 2.0)

*Missing a valid response: IFA: 21, diarrhoea management: 7, birth spacing: 9
† All models adjusted for age, caste, religion and birth order
‡ p-value = 0.05
